# Correlation of myocardial perfusion defects on Stress MR perfusion study with occurrence major adverse cardiac events in patients with moderate coronary artery disease

**DOI:** 10.1186/1532-429X-17-S1-P171

**Published:** 2015-02-03

**Authors:** Mahesha Bannur

**Affiliations:** Radiology, Vikram Hospital Mysore, Mysore, India; Radiology, Diplomate National Board, New Delhi, India

## Background

Is to establish the relationship between Cardiac Stress MR Perfusion findings with occurrence of major adverse cardiac events in patients with moderate coronary artery stenosis.

## Methods

Performed stress CMR for 80 patients referred for ischemia assessment after Invasive Coronary angiogram. CMR examination included are Cine Cardiac function , Adenosine stress Perfusion, Rest Perfusion and late Gadolinium enhancement with appropriate sequences.

Follow up done for these Patients for a period of one year after CMR study for occurrence of Major adverse cardiac events (MACE - Myocardial Infarction (MI), Unstable angina, Arrhythmias & Death).

## Study Period

June 2010 to Sep 2013 . MR equipment - 1.5 Tesla ( Avanto-Siemens).

Adenosine used as Stress agent & Contrast is Gadolinium based.

## Data Analysis

Stress MR - Dark / Hypo intense defect in the Myocardial wall of the arterial territory.

Major adverse Cardiac Events - Follow up Patients done for a period of one year after CMR study, for occurrence of Myocardial Infarction (MI), Unstable angina, Arrhythmias & Death.

## Results

80 patients with moderate CAD.

Stress CMR > Perfusion defects in 54 patients & No defects in 26 patients.

54 Patients > 32 had Transmural & 22 had Subendocardial perfusion defects in corresponding territories.

Follow up for MACE:

29 of 80 Patients with moderate CAD (29%) had adverse events & 28 of 54 patients with CMR -Perfusion defects had adverse events ( 51 %).

19 of 32 Transmural Perfusion defects had adverse events - 7 had MI , 6 had unstable angina, 4 arrthymia & 2 deaths (59% MACE).

9 of 22 Subendocardial Perfusion defects had adverse events - 2 had MI , 4 Unstable angina,& 3 arrthymia (40% MACE).

1 of 26 patients without CMR- Perfusion defect had unstable angina in follow period.

## Conclusions

Stress perfusion CMR study will assess the Significance of moderate Coronary artery stenosis for Inducible Ischemia by detecting the perfusion defects.

Development of Adverse cardiac events is more likely in patients with moderate CAD demonstrating Perfusion defects on stress CMR.

## Funding

No funding.

